# Adherence to heart rate-based intensity parameters predicts cardiovascular response to 12-weeks of aerobic cycling training in sedentary older adults

**DOI:** 10.1016/j.pmedr.2026.103388

**Published:** 2026-01-21

**Authors:** Tom S. Novak, Caroline Quan, Keith McGregor, Kevin Mammino, Medina Bello, Joe R. Nocera

**Affiliations:** a Center for Visual and Neurocognitive Rehabilitation (CVNR), Atlanta VAMC, Decatur, GA, United States; b Division of Physical Therapy, School of Medicine, Emory University, Atlanta, GA, United States; c School of Medicine – Center for Clinical and Translational Science (CCTS), The University of Alabama at Birmingham, Birmingham, AL, United States

**Keywords:** Exercise adherence, Exercise intensity, Heart-rate reserve (HRR), Aerobic exercise, Aging

## Abstract

**Objectives:**

Exercise improves cardiovascular health in older adults, yet variability in responsiveness remains. This study tested if prescribed exercise intensity adherence predicts individual differences in cardiovascular fitness gains following an intervention.

**Methods:**

From 2017 to 2022, nineteen sedentary older adults (69.78 ± 6.47 years) completed a 12-week aerobic cycling program in the Exercise Research Laboratory at the Atlanta VA Hospital. Participants trained 3× weekly, progressing from 20 to 45 min per session. Target intensity, expressed as percent heart rate reserve (HRR), increased from 50% to 80%. Cardiovascular fitness ($\dot{V}O$_2max_) was estimated pre- and post-intervention using the YMCA submaximal test. Attendance reflected the proportion of completed sessions. Intensity-based adherence was derived from the ratio of median HR to prescribed HR across sessions, modeled over time, and expressed as change from session 1 to 36.

**Results:**

Estimated $\dot{V}O$_2max_ increased significantly (21.51 ± 8.77 to 25.80 ± 9.42 ml/kg/min, *p* < .001). Attendance exceeded 90% and did not predict estimated $\dot{V}O$_2max_ change, whereas HR adherence to prescribed intensity significantly predicted improvement (R^2^ = 0.42, *p* = .01).

**Conclusions:**

Cardiovascular adaptations in older adults relate more strongly to prescribed HR adherence than attendance. Objective, trajectory-based adherence metrics may refine exercise prescriptions and improve prediction of individual responsiveness.

## Highlights

• Cycling training improved VO2max by ∼20% over 12 weeks.
• Intensity adherence, not attendance, predicted VO2max gains in older adults.
• Polynomial adherence trends explained 42% of VO2 variance.

## 1 Introduction

Over the last half-century, ample research has established exercise as one of the most impactful interventions for human health and function. Guidelines developed by the World Health Organization (WHO) suggest that adults who achieve 150–300 min of moderate-intensity exercise, or 75–150 min of aerobically vigorous exercise per week can enjoy beneficial effects to their central, cardiovascular, and musculoskeletal systems (Organization WH, 2019; World Health Organization, 2010), along with improvements in overall cognition and emotional health (Kim et al., 2022).

Despite systemic improvements reported consistently at the group level, variability in individualized response to exercise remains prevalent (Chrzanowski-Smith et al., 2020; Vellers et al., 2018; Williamson et al., 2017). A key avenue of study is to understand factors contributing to this variability and optimize response outcomes in individuals who show minimal or even negative changes in their functional and cardiovascular health (non-responders and adverse responders, respectively). Exercise frequency, duration, and intensity play a significant role in driving systemic training adaptations (i.e., cardiovascular fitness, force production, muscular endurance, improved balance), and thus careful selection and titration of these parameters is critical to maximizing individual dose response. While intervention studies adopt these principles to maximize physiological challenge and minimize factors of attrition (i.e., fatigue, boredom), differential response to exercise remains prevalent, especially in the context of aging (Kelley et al., 2024; Herold et al., 2021; Cano-Montoya et al., 2025).

To better account for this variability, objective measures of exercise adherence have been developed to assess how fidelity to individual parameters of exercise prescription predict response to exercise. For example, studies on middle-aged adults have found that adherence to weekly, prescribed minutes of exercise improved outcomes of bodily pain, cardiovascular fitness, and physical function (Imayama et al., 2013). Moreover, aging studies have demonstrated an association between lower attendance rates and higher incidence of “non-response” to exercise in terms of cardiovascular fitness and clinically meaningful weight loss outcomes (Fielding et al., 2007; Whipple, 2019; Van Gool et al., 2005). However, this relation is less clear in studies with low intensity-based adherence due to high overall attendance rates (>80%) (Chmelo et al., 2015; Swift et al., 2016).

While these findings highlight the value of understanding prescribed exercise intensity adherence, this literature is limited in that a) adherence is generally examined according to a single dimension of exercise prescription (attendance or duration of total minutes), and b) these adherence measures do not adequately assess fidelity to prescribed intensity of exercise, as measured by target HR, for example. Miller et al., (2014) (Miller et al., 2014) addressed these limitations in young healthy adults by deriving heart rate-based physical activity scores (HRPAS) formulated according to individuals' total adherence (duration and intensity) over a 15-week aerobic intervention. They found that higher HRPAS scores were associated with significant positive improvements in body composition (BMI, body fat percentage, waist/hip circumference) and outcomes of cardiometabolic health (systolic blood pressure, fasting glucose, resting heart rate, total cholesterol).

In the present study, we examined the relationship between adherence to prescribed exercise intensity (heart rate (HR) adherence) during a 12-week exercise intervention and improvements in cardiovascular fitness, measured by estimated $\dot{V}O$_2max_, in sedentary older adults (65–80 years). Because HR adherence can vary across training as participants adapt to repeated exercise and experience fluctuations in fatigue, recovery, and motivation, we modeled each participant's adherence trajectory across the full intervention and tested whether these trajectories predicted inter-individual variability in estimated $\dot{V}O$_2max_ improvement. We hypothesized that individual differences in adherence to a progressively titrated intensity prescription, captured across time, would explain variability in training response as indexed by change in estimated $\dot{V}O$_2max_

We evaluated a supervised aerobic cycling program in which exercise frequency was held constant, exercise duration increased at the same rate over time, and exercise intensity was progressively advanced using targets based on heart rate reserve (HRR). We also examined attendance-based adherence (number of sessions attended), a commonly used metric, in relation to changes in estimated $\dot{V}O$_2max_. We hypothesized that HR adherence would explain inter-individual variability in fitness improvements more effectively than attendance alone. Accordingly, participants wore HR monitors during every session to quantify session-specific HR adherence to the prescribed intensity targets.

## 2 Methods

Prior to any testing procedures, study personnel provided detailed explanations of any potential risks of the experiment and attained informed consent with all participants. All study ethics and procedures complied with the Declaration of Helsinki and were approved by the Emory University Internal Review Board and Atlanta VA Research and Development office (IRB00059193, IRB00056726).

### 2.1 Study design and population

All participants self-reported as sedentary according to the American College of Sports Medicine's (ACSM) criteria (< 120 min/week). Inclusion criteria required that participants a) be between 65 and 80 years of age, b) have no prior history of psychiatric illness or neurological disease, and c) attain physician clearance indicating that they can safely participate in all graded exercise testing and exercise intervention procedures. Exclusionary factors included a) any conditions that would contraindicate graded exercise testing, b) failure to provide informed consent, c) hospitalization within the past 6 months, d) inability to walk at least 400 m unassisted, and e) uncontrolled hypertension and/or diabetes.

#### 2.1.1 Exercise intervention

Upon attaining informed consent, participants were enrolled into a supervised aerobic cycling intervention conducted at the Atlanta VA Medical Center from 2017 to 2022. Participants completed guided exercise three times per week for 12 weeks, with all sessions supervised by a trained interventionist. The aerobic intervention consisted of interval-based training on a stationary bike designed to improve aerobic capacity and muscular endurance.

#### 2.1.2 Session structure and progression

**Fig. 1:**
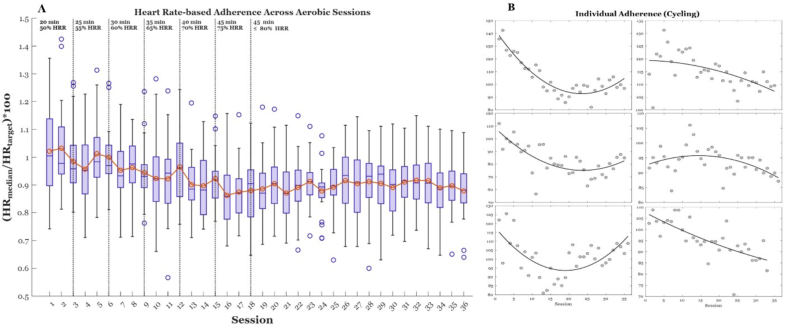
(2 A) Box-plot illustration of group-level heart rate adherence across 12 weeks (36 sessions) of prescribed aerobic cycling conducted at the Atlanta VA Medical Center between 2017 and 2022. Nineteen sedentary older adults completed the cycling intervention; however, heart rate adherence is reported for 17 participants (11 Female) due to heart rate signal dropout during exercise in two participants. Each box-and-whisker depicts adherence within a session, expressed as the ratio of participants' median within-session heart rate to the prescribed target heart rate for that session. Dotted vertical lines indicate intervals where exercise duration and intensity (%HRR) were titrated. (2B) Exemplary individual-level heart rate adherence data and fitted adherence trends (2nd-order polynomial) for six participants.

To begin the intervention, sessions consisted of 20 min of continuous cycling. Session duration was progressively increased by 1–2 min such that the first session of each week was 5 min longer than the week before. Duration continued to increase until participants reached 45 min of exercise (session 15), which was then maintained for all remaining sessions. Exercise intensity was progressively titrated over the course of the intervention from 50% to 80% HRR by week 6 (18 sessions) (see Fig. 1). Participants were then instructed to maintain 80% HRR for the remainder of the intervention.

#### 2.1.3 Heart rate monitoring and supervision

During all sessions, participants wore chest-strap heart rate monitors (Polar H1) paired with watches (Polar FT7; Polar Electro OY, Kempele, Finland). Instructors manually recorded heart rate every 5 min for the duration of each session. At the same time heart rate values were recorded, the supervising interventionist also documented participants' verbalized rate of perceived exertion (RPE). The interventionist provided continuous feedback to participants regarding whether they were maintaining their target heart rate for that session. If participants fell above or below five beats per minute of their prescribed target, the interventionist instructed them to increase or decrease their pedaling intensity until they reached or exceeded the target heart-rate range. Attendance and HR were recorded throughout the intervention.

#### 2.1.4 Determining and prescribing target exercise intensity

Target heart-rate zones were calculated using the Karvonen method for estimating HRR:

(1) $${\mathrm{HR}}_{\mathrm{reserve}}={\mathrm{HR}}_{\max}-{\mathrm{HR}}_{\mathrm{resting}}$$

*HR*_*max*_ was estimated as *220 − age*. Resting heart rate was collected during the baseline visit while participants sat quietly for 1 min. Participants' individualized target heart rate for each session was then calculated using:

(2) $${\mathrm{HR}}_{\mathrm{target}}=\left({\mathrm{HR}}_{\mathrm{reserve}}\%\mathrm{intensity}\right)+{\mathrm{HR}}_{\mathrm{resting}}$$

Here, *% intensity* reflects the prescribed percentage of HRR, which progressed from 50% up to 80% HRR as described above.

#### 2.1.5 Estimated $\dot{V}O$_2max_ assessment

Participants underwent graded exercise testing within 2 weeks of the first and last session of the exercise intervention. Cardiovascular fitness was assessed via a YMCA submaximal fitness test using a cycle ergometer (Monark Ergomedic 839 E, Vansbro, Sweden). This test extrapolates participants' maximal rate of oxygen uptake based on changes to their heart rate during up to 4 stages of exercise (3 min/stage) at graded workloads. The Monarch ergometer expresses workload as the product of resistance load (in kilograms) and distance traveled (in meters per revolution).

Participants performed a 2-min warm up where they pedaled at a self-selected pace and zero resistance. Stage 1 of the test was then initiated, where participants pedaled at a workload of 0.5 kg ⸳ m/min for 3-min. Average steady-state heart rate was calculated from stage 1 and used to assign a specific workload for stage-2 (<80 bpm: 2.5 kg ⸳ m/min, 80–89 bpm: 2.0 kg ⸳ m/min, 90–100 bpm: 1.5 kg ⸳ m/min, >100 bpm: 1.0 kg ⸳ m/min). Workload was then increased in *0.5* kg ⸳ m/min increments for every subsequent exercise stage until participants reached 85% of their estimated heart-rate maximum (220-age*0.85). From these data, participants $\dot{V}O$_2max_ was calculated based on the following steps: a) Participants work rate data was used to estimate increases in metabolic energy expenditure ($\dot{V}O$_2_) based on ACSM's equation for cycling: $\dot{V}O$_2_ = 1.8 * (work rate(kg ⸳ m/min) / body mass(kg)) + 3.5. b) Least-squares regression was then used to fit change in heart rate as a function of change in these $\dot{V}O$_2_ estimates. c) The extrapolated $\dot{V}O$_2_ value corresponding to participants' estimated heart rate max (220-age) was then used as our estimated $\dot{V}O$_2max_

### 2.2 Measures

#### 2.2.1 Attendance

Attendance was expressed as the percentage of sessions attended (sessions attended / 36).

#### 2.2.2 Prescribed HR adherence

HR was manually recorded every 5 min during each session of the 12-week intervention. These data were compiled in Excel and processed using custom MATLAB scripts to compute adherence according to Eq. 3:

(3) $$\mathrm{HR}\;\mathrm{Adherence}={\left({\mathrm{HR}}_{\mathrm{median}}/{\mathrm{HR}}_{\mathrm{target}}\right)}^{*}\;100$$

where *HR*_*median*_ represents the within-session median HR and *HR*_*target*_ represents the prescribed target HR for that session (derived from Eq. 2). Target intensity increased weekly until week 6 and remained constant thereafter. Group- and individual-level prescribed HR adherence trajectories are shown in Fig. 1.

#### 2.2.3 Modeling HR adherence across the intervention

Because HR adherence can fluctuate across sessions due to routine biological and measurement variability, we used polynomial fitting as a preprocessing step to smooth day-to-day noise and derive participant-specific adherence trajectories across the intervention, thereby providing a stable estimate of each participant's underlying adherence pattern without allowing any single session to disproportionately influence the summary measure; this trajectory-based approach also aligns with our primary aim of determining whether participants with improving, deteriorating, or nonlinear adherence patterns differ in their $\dot{V}O$_2_ response, as trajectories capture direction and curvature that a grand-mean adherence value cannot. We compared 1st-, 2nd-, and 3rd-order polynomial models and selected the most stable and parsimonious fit for trend derivation, with model comparison statistics reported in Supplementary Table S1.

#### 2.2.4 Change in HR adherence

Using the selected model (2nd-order), we derived smoothed Day 1 and Day 36 adherence estimates for each participant. These model-derived values were used to compute the adherence indices reported in Table 1, including Day 1, Day 36, absolute change (Day 36 − Day 1), and proportional change [(Day 36 − Day 1)/Day 1]. Change in HR adherence was defined as the proportional change from Day 1 to Day 36 using these smoothed estimates. This approach accounts for baseline differences in HR adherence and avoids penalizing participants whose absolute HR adherence values were high throughout training.

**Table 1:** Individual-subject changes in estimated $\dot{V}O$_2max_, attendance, and adherence to prescribed target heart rate across 12 weeks of supervised aerobic cycling conducted at the Atlanta VA Medical Center between 2017 and 2022. These data were collected from a cohort of 19 sedentary older adults (13 Female) from the Greater Atlanta Area (USA). Heart rate adherence is reported for 17 participants (11 Female) due to heart rate signal dropout during exercise in two participants.

Subject	∆ Estimated $\dot{V}O$_2max_ (ml/kg/min)	Attendance (%)	HR Adherence (% Target HR)			
			Day 1	Day 36	∆ (Day 36 - Day 1)	∆ (Day 36 - Day 1) / Day 1
01	1.30	100	93.26	81.31	−11.95	−0.13
02	3.80	100	119.43	107.33	−12.10	−0.10
03	4.40	90	87.79	80.19	−7.60	−0.09
04	4.74	90	105.88	94.56	−11.32	−0.11
05	5.20	90	96.86	80.15	−16.71	−0.17
06	6.70	90	97.92	93.00	−4.92	−0.05
07	2.20	86	106.73	86.26	−20.47	−0.19
08	2.68	97	138.39	104.76	−33.63	−0.24
09	3.09	97	126.73	–	–	–
10	2.20	100	110.33	–	–	–
11	5.89	100	82.45	67.01	−15.44	−0.19
12	0.99	100	86.79	66.36	−20.43	−0.24
13	3.50	90	100.93	97.51	−3.42	−0.03
14	9.34	95	91.36	87.86	−3.50	−0.04
15	1.45	92	99.31	88.67	−10.64	−0.11
16	5.00	92	115.20	112.87	−2.33	−0.02
17	0.93	92	99.81	86.71	−13.10	−0.13
18	4.70	72	105.62	82.70	−22.92	−0.22
19	13.23	97	92.93	97.93	5.00	0.05
**Group: Mn (±Std)**	**4.28** **(±2.98)**	**93.21** **(±6.64)**	**103.04** **(±13.87)**	**89.13** **(±12.41)**	**−12.09** **(±8.96)**	**−0.12** **(±0.08)**

Table values are reported for each participant and include ∆ in estimated $\dot{V}O$_2max_ (post − pre), attendance (% of 36 sessions), and prescribed HR adherence (% target HR) at Day 1 and Day 36. HR adherence was calculated as the ratio of within-session median HR to session-specific target HR × 100. To reduce day-to-day fluctuations and derive adherence trends, session-level adherence was smoothed using the selected 2nd-order polynomial model; accordingly, Day 1, Day 36, absolute change (Day 36 − Day 1), and proportional change [(Day 36 − Day 1)/Day 1] reflect model-derived smoothed estimates. Two participants completed the intervention but were missing Day 36 HR adherence values due to dropout of heart rate recordings acquired during exercise.

**Table 2:** Multiple regression model predicting change in estimated $\dot{V}O$_2max_ from attendance and change in HR adherence during a 12-week cycling intervention conducted at the Atlanta VA Medical Center between 2017 and 2022. Analysis includes 17 sedentary older adults (11 Female) from the Greater Atlanta Area (USA).

Parameter	β	Std. Err	t	*p*	Variance Inflation Factor (VIF)
Intercept	9.88	9.09	1.09	0.29	.
**HR Adherence** **∆ (Day 36 - Day 1) / Day 1**	**24.21**	**7.99**	**3.03**	0.01*	**1.02**
Attendance (%)	−0.03	0.09	−0.29	0.77	1.02

The dependent variable was ∆ estimated *VO*_2max_ (ml/kg/min). Predictors included attendance (% of sessions attended) and change in prescribed HR adherence expressed as proportional change from Day 1 to Day 36. Adherence values used to derive the change metric were based on smoothed adherence trajectories generated using the selected 2nd-order polynomial model. β denotes the model coefficient, Std. Err denotes the standard error of model coefficients, t denotes the t-statistic, p denotes the alpha threshold, VIF denotes the variance inflation factor testing for collinearity (1.0 = zero collinearity, >5 significant collinearity). * indicates significance at *p* ≤ .05. Text in bold indicates statistical significance.

### 2.3 Statistical analysis

#### 2.3.1 Intervention effect

We examined the effects of the 12-week aerobic cycling intervention on cardiovascular fitness. Changes in estimated $\dot{V}O$_2max_ were evaluated using a paired-samples *t*-test comparing pre- and post-intervention estimated $\dot{V}O$_(2max)_

#### 2.3.2 Predicting estimated $\dot{V}O$_2max_ change based on attendance and HR adherence

Changes in estimated $\dot{V}O$_2max_ were evaluated using a paired-samples t-test comparing pre- and post-intervention values.

#### 2.3.3 Predicting estimated $\dot{V}O$_2max_ change based on attendance and HR adherence

Multiple regression analysis was used to evaluate the degree to which changes in estimated $\dot{V}O$_2max_ were accounted for by attendance and change in prescribed HR adherence. The fitted model is shown in Eq. 4:

(4) $$\mathrm{\Delta Estimated}\;V˙O_{2\max}=A+B\;\mathrm{HRAdherenc}e_{\Delta \left(\mathrm{Day}36–\mathrm{Day}1\right)/\mathrm{Day}1}+C\;\mathrm{Attendance}+e$$

where *A, B, C* represent fitted parameters and *e* is the error term.

## 3 Results

### 3.1 Intervention effects

We report on data collected from 19 healthy volunteers from the Greater Atlanta area (69.78 ± 6.47 years, 13 Female). Individual-subject attendance, prescribed HR adherence values, and changes in estimated $\dot{V}O$_2max_ are presented in Table 1. Group-level attendance was 93.21% (± 6.64), indicating high participation across the 36-session intervention.

**Fig. 2:**
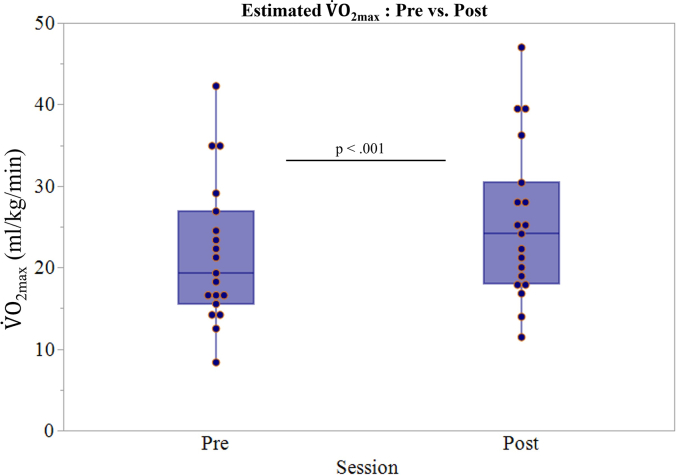
Boxplot illustration of change in estimated $\dot{V}O$_2max_ after the 12-week guided aerobic cycling intervention conducted at the Atlanta VA Medical Center between 2017 and 2022. $\dot{V}O$_2max_ data was collected from a cohort of 19 sedentary older adults (13 Female) from the Greater Atlanta Area (USA).

Estimated $\dot{V}O$_2max_ significantly increased following 12 weeks of cycling (pre: 21.51 ± 8.77 ml/kg/min; post: 25.80 ± 9.42 ml/kg/min; *p* < .001). The group-level ∆ estimated $\dot{V}O$_2max_ was 4.28 (± 2.98) ml/kg/min, reflecting a substantial improvement in cardiovascular fitness with clear inter-individual variability (Fig. 2).

Two female participants completed the intervention, however, there were issues with dropout in HR data during exercise at higher intensities (after week 6). As such, these participants were excluded from analyses requiring end-of-intervention adherence metrics.

### 3.2 Prescribed HR adherence patterns

Group-level prescribed HR adherence values are summarized in Table 1. Adherence values in Table 1 (Day 1, Day 36, absolute change, and proportional change) were derived from smoothed adherence trajectories generated using the selected 2nd-order polynomial model.

Mean HR adherence declined from Day 1 (103.04 ± 13.87% target HR) to Day 36 (89.13 ± 12.41% target HR), corresponding to an average absolute change of −12.09 (± 8.96) and proportional change of −0.12 (± 0.08). This pattern likely reflects the progressive increase in prescribed intensity early in the intervention and the challenge of maintaining higher targets across later training weeks. At the individual level, variability in these values suggests that some participants maintained or improved intensity fidelity while others exhibited more pronounced declines.

### 3.3 **Predicting estimated**$\dot{V}O$_2max_**change based on attendance and adherence**

To examine whether intensity-based adherence accounted for inter-individual variability in fitness gains beyond session exposure, we modeled ∆ estimated $\dot{V}O$_2max_ as a function of attendance and change in HR adherence. Analyses requiring Day 36 adherence values included 17 participants.

**Fig. 3:**
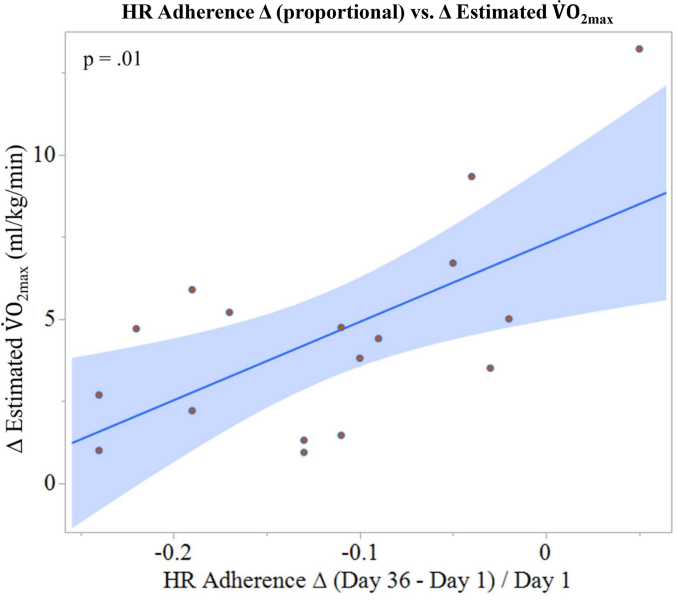
Least squares regression illustrating the association between proportional change in prescribed HR adherence and change in estimated $\dot{V}O$_2max_ following 12 weeks of supervised aerobic cycling. Data are shown for 17 sedentary older adults (11 Female) from the Greater Atlanta Area (USA). HR adherence change was calculated as proportional change from Day 1 to Day 36 $\left(\mathrm{Day}36-\mathrm{Day}1\right)/\mathrm{Day}1$, using smoothed adherence estimates (see Table 1). This figure is provided for illustration of the primary adherence–fitness relationship and reflects a bivariate depiction of the adherence predictor included in the multiple regression model. As reported in Table 2, the multiple regression model included both attendance and HR adherence change as independent variables and demonstrated that HR adherence change accounted for significant variance in ∆ estimated $\dot{V}O$_2max_ after training, whereas attendance did not.

The multiple regression model was statistically significant, F (2, 14) = 4.59, *p* = .03, accounting for 42% of the variance in estimated $\dot{V}O$_2max_ change (Fig. 3). When controlling for both predictors, change in HR adherence was a significant contributor (β = 24.21, *t* = 3.03, *p* = .01). Attendance was not a significant predictor of estimated $\dot{V}O$_2max_ improvement (β = −0.03, *t* = −0.29, *p* = .77), and collinearity was minimal (VIFs = 1.02).

Collectively, these findings indicate that changes in physiological engagement with prescribed intensity over time were more strongly related to fitness adaptation than session completion alone. This supports the central premise that high attendance does not necessarily translate into uniform cardiovascular benefit when intensity-based fidelity varies across individuals.

## 4 Discussion

This study sought to investigate the relationship between adherence to prescribed exercise intensity and individual variability in cardiovascular fitness outcomes among sedentary older adults following a 12-week supervised exercise intervention. Participants completed an aerobic cycling intervention with matched frequency and progressive duration, and with a systematically increasing intensity profile. Our findings demonstrated that aerobic cycling training led to significant improvements in estimated $\dot{V}O$_2max_. Importantly, only HR adherence to prescribed exercise intensity, quantified by a dynamic, session-by-session heart rate tracking approach, was significantly associated with improvements in $\dot{V}O$_2max_. Whereas attendance, a commonly used metric of overall adherence, was not statistically related to change in $\dot{V}O$_2max_. These findings provide strong evidence that exercise intensity is a primary determinant of cardiorespiratory fitness adaptation in older adults and underscore the importance of individualized, physiologically-based adherence metrics in explaining the variability of intervention outcomes.

While it is well established that aerobic exercise can promote cardiovascular and metabolic health in aging populations (Booth et al., 2011; Seals et al., 2016), our findings reinforce that these benefits are not merely a function of participation or time spent exercising but rather depend critically on the fidelity with which prescribed intensity is followed. This aligns with studies such as that by Swain and Franklin (2006) (Swain and Franklin, 2006), which emphasized that moderate- to high-intensity aerobic exercise leads to greater increases in $\dot{V}O$_2max_ and associated cardioprotective effects compared to lower intensity exercise. Our results extend this line of evidence by showing that even within a structured intervention with high attendance rates, only participants who consistently adhered to rising intensity prescriptions exhibited marked physiological improvements.

These findings parallel those reported in the TIGER study (Miller et al., 2014), which developed a heart rate–based physical activity score (HRPAS) in a younger adult population. The HRPAS captured adherence to both the duration and intensity of exercise and was significantly associated with improvements in blood pressure, fasting glucose, and lipid profiles. Our approach adds to this work by modeling adherence as a dynamic process using session-level heart rate data and polynomial fitting to track changes in fidelity to intensity over time. Rather than averaging heart rate adherence over weeks or months, our method allowed us to capture nonlinear trends that may reflect physiological adaptation, behavioral fatigue, or lapses in engagement. This modeling showed that the session-by-session pattern of adherence across the 12-week intervention, including both declines and recovery periods, was a strong predictor of individual $\dot{V}O$_2max_ improvements.

Interestingly, our results suggest that attendance alone, while important for assessing intervention feasibility and compliance, is insufficient for explaining who benefits from exercise training. Although participants demonstrated exceptionally high attendance (>90%), no relationship was found between session frequency and changes in $\dot{V}O$_2max_. This mirrors findings from Whipple et al. (2019) (Whipple, 2019), who reported that attendance rates were not associated with gains in physical function or $\dot{V}O$_2_ among older adults when variability in attendance was low. Similarly, in a large, randomized trial by Fielding et al. (2007) (Fielding et al., 2007), adherence to physical activity guidelines was necessary but not sufficient for producing consistent metabolic benefits, indicating that other factors including exercise dose and physiological engagement are likely more important determinants of response.

This study also highlights a critical methodological issue in exercise adherence research: most studies quantify adherence in a unidimensional manner, typically by tracking attendance or total minutes of participation. These metrics, while easy to collect and report, fail to reflect whether the participant actually engaged with the exercise at the prescribed intensity. For interventions where physiological challenge is central to the targeted outcomes, such as $\dot{V}O$_2max_, such misalignment may obscure true dose-response relationships. Our intensity-based metric, which tracked heart rate adherence over time using individualized heart HRR targets, offers a more accurate lens into the participant's physiological engagement during the intervention.

The application of wearable technologies presents promising future directions. Devices capable of continuously recording heart rate, energy expenditure, and other biometric parameters offer high-resolution insight into the temporal dynamics of exercise adherence. With more granular data, researchers and clinicians could better detect early warning signs of plateau, fatigue, or disengagement, and adapt intervention protocols accordingly. By modeling adherence trajectories in real-time, clinicians may be able to stratify participants by risk of non-response and personalize feedback to maximize the probability of benefit.

There are several limitations to our study. First, the relatively small sample size may have reduced our ability to detect smaller but meaningful effects and limits the generalizability of our findings. Second, although we used a validated submaximal protocol to estimate $\dot{V}O$_2max_, direct measurement via metabolic cart would provide more precise quantification of cardiorespiratory capacity. Third, estimation of heart rate maximum using 220-age has been shown to underestimate individuals true maximum heart rate in older age groups. However, because we applied this method uniformly across participants to determine targeted exercise heart rates, and these target heart rates still fall well within those recommended to elicit cardiovascular adaptations, we do not believe this adversely affected our study findings. Fourth, our adherence modeling was based solely on heart rate, which, while appropriate for aerobic interventions, may not effectively capture exertion during non-aerobic or neuromotor training. In such contexts, changes in neuromuscular coordination, joint stability, or postural control might occur despite the absence of $\dot{V}O$_2max_ changes. Future studies incorporating force platforms, electromyography (EMG), or wearable inertial measurement units (IMUs) may help quantify intensity in non-aerobic modalities more effectively (Granacher et al., 2011; Pijnappels et al., 2001).

## 5 Conclusion

This study emphasizes that individualized fidelity to prescribed exercise intensity, rather than simple participation, is a critical determinant of cardiovascular adaptation in older adults. By dynamically modeling adherence over time using heart rate data, we were able to explain significant inter-individual variability in response to aerobic cycling training. These findings advocate for the expanded use of multi-dimensional adherence metrics, supported by wearable technologies, to personalize exercise interventions and optimize outcomes in aging populations.

The authors declare that the research was conducted in the absence of any commercial or financial relationships that could be construed as a potential conflict of interest.

TN contributed to the original drafting of this manuscript, data processing, statistical analysis, and manuscript revision after original drafting. CQ contributed to the original drafting of this manuscript, data processing, and manuscript revision after original drafting. KMcG contributed to study conceptualization, original drafting of this manuscript, data collection, and manuscript revision after original drafting. KM contributed to study conceptualization, original drafting of this manuscript, data collection, data processing, and manuscript revision after original drafting. MB contributed to original drafting of this manuscript, data collection, and manuscript revision after original drafting. JN contributed to study conceptualization, original drafting of this manuscript, data collection, and manuscript revision after original drafting. TN and CQ contributed equally to this manuscript as co-first authors. All authors reviewed and provided final approval of the manuscript prior to resubmission.

## Credit authorship contribution statement

**Tom S. Novak:** Writing – review & editing, Writing – original draft, Visualization, Validation, Methodology, Investigation, Formal analysis, Data curation. **Caroline Quan:** Writing – review & editing, Writing – original draft, Visualization, Validation, Methodology, Formal analysis, Data curation. **Keith McGregor:** Writing – review & editing, Supervision, Resources, Project administration, Methodology, Investigation, Funding acquisition, Conceptualization. **Kevin Mammino:** Writing – review & editing, Validation, Methodology, Data curation, Conceptualization. **Medina Bello:** Writing – review & editing, Project administration, Methodology, Investigation, Data curation. **Joe R. Nocera:** Writing – review & editing, Writing – original draft, Supervision, Resources, Project administration, Methodology, Investigation, Funding acquisition, Data curation, Conceptualization.

## Declaration of competing interest

The authors declare that they have no known competing financial interests or personal relationships that could have appeared to influence the work reported in this paper.

## Data Availability

Data will be made available on request.

## Appendix A Supplementary data

Supplementary material Image 1
