# Supplementary material for: Adherence to heart rate-based intensity parameters predicts cardiovascular response to 12-weeks of aerobic cycling training in sedentary older adults
Source: Prev Med Rep. 2026 Jan 21;62:103388. doi: 10.1016/j.pmedr.2026.103388 (PMC12874327; doi:10.1016/j.pmedr.2026.103388)
Supplement: Supplementary file 1 — Supplementary material [file mmc1.docx]

| **Polynomial Fitting** | ***Adjusted R^2^*** | **AIC** | **BIC** |
| --- | --- | --- | --- |
| 1^st^ Order | .19 (.17) | 523.15 (115.32) | 521.15 (115.32) |
| 2^nd^ Order | .28 (.20) | 517.17 (113.75) | 515.18 (113.75) |
| 3^rd^ Order | .29 (.21) | 505.04 (158.40) | 503.04 (158.04) |

**Supplementary Table 1.** Results from polynomial fitting comparisons on individual-level trends in heart rate-based adherence for participants.

Mean (SD) adjusted R², Akaike Information Criterion (AIC), and Bayesian Information Criterion (BIC) values are shown for 1st-, 2nd-, and 3rd-order polynomial models fit to participants’ session-level HR adherence across the intervention. Adjusted R² reflects goodness-of-fit while penalizing added model complexity, whereas lower AIC/BIC values indicate better relative model fit with penalties for overfitting (stronger for BIC), supporting selection of the most parsimonious adherence trend model.
